# Numerical Modelling of the Ultrasonic Treatment of Aluminium Melts: An Overview of Recent Advances

**DOI:** 10.3390/ma12193262

**Published:** 2019-10-06

**Authors:** Bruno Lebon, Iakovos Tzanakis, Koulis Pericleous, Dmitry Eskin

**Affiliations:** 1 Brunel Centre for Advanced Solidification Technology, Brunel University London, Kingston Lane, Uxbridge UB8 3PH, UK; 2 Oxford Brookes University, Wheatley Campus, Oxford OX33 1HX, UK; itzanakis@brookes.ac.uk; 3 Computational Science and Engineering Group, University of Greenwich, 30 Park Row, London SE10 9LS, UK; K.Pericleous@greenwich.ac.uk

**Keywords:** numerical modelling, acoustic cavitation, aluminium, ultrasonic melt treatment, non-linear bubble dynamics, sonoprocessing

## Abstract

The prediction of the acoustic pressure field and associated streaming is of paramount importance to ultrasonic melt processing. Hence, the last decade has witnessed the emergence of various numerical models for predicting acoustic pressures and velocity fields in liquid metals subject to ultrasonic excitation at large amplitudes. This paper summarizes recent research, arguably the state of the art, and suggests best practice guidelines in acoustic cavitation modelling as applied to aluminium melts. We also present the remaining challenges that are to be addressed to pave the way for a reliable and complete working numerical package that can assist in scaling up this promising technology.

## 1. Introduction

Process design has the potential to provide a strategic competitive advantage with regards to customer appeal, product cost and innovation [1,2]. A key element of process innovation involves a fundamental understanding of how materials and process interactions determine manufacturing performance [2].

A continuous mode of production is often more desirable than batch production. Advantages of continuous operations include cheaper unit costs of production, energy savings and homogenization in the quality of the product. However, converting batch processes to continuous modes is not straight-forward [3]. In the past six years, the authors have been researching how to upscale the promising technology of ultrasonic melt processing by moving applications from batch mode to inline mode [4].

Ultrasonic melt processing (USP) is an effective method for degassing, filtration and grain refinement of light metal alloys on the industrial scale [5,6,7]. The beneficial effects of USP are attributed to acoustic cavitation—the violent pulsation and collapse of gas bubbles under the influence of a strong acoustic pressure field [8,9], and acoustic streaming—the fluid motion that results from the attenuation of the acoustic pressure wave as it propagates in the liquid [10]. While USP works well in batch degassing or grain refining of a single cast billet or ingot in direct-chill (DC) casting [11], it does not scale up very well for continuous processing, unless multiple ultrasound sources are used [4]. Current research is now focusing on upscaling this promising technology and achieving high efficiency when treating large melt volumes in continuous mode and with a minimum number of ultrasound sources.

To optimize USP, recirculation patterns and mass exchanges between the cavitation zone and the rest of the liquid bulk need to be adequately quantified. In addition, melt recirculation reduces temperature gradients and promotes a preferred equiaxed grain structure [12]. In DC casting, acoustic streaming improves chemical homogeneity and promotes grain refinement through deagglomeration of clusters, wetting of inclusions, dispersion of substrates and solid-phase fragmentation [7,13,14]. However, it is challenging to visualize acoustic streaming in liquid aluminium due to its opaqueness and high operating temperature. Therefore, studies of acoustic streaming must include modelling in conjunction with X-ray imaging [15,16,17,18] to predict the generation of cavitation bubbles, their transport and acoustic propagation in the presence of attenuation.

A recent review of acoustic pressure modelling presents the challenges facing acoustic cavitation modelling [19] and has been the starting point for the model that resulted from a joint collaboration between Brunel and Greenwich universities in the U.K. [4]. Since then, a novel ‘advanced’ model that incorporates acoustic streaming with cavitation dynamics has been made available [20], enabling more accurate predictions of processing simulations involving cavitation bubbles at an affordable computational cost. This recent progress is summarised in this overview giving readers a good starting point in ultrasonic process modelling.

## 2. Existing Models

### 2.1. Acoustic Cavitation Model

The theory is reproduced here as a comprehensive summary of acoustic cavitation treatment in modelling of ultrasonic melt processing. The starting point of acoustic cavitation modelling is the Caflisch equations [21]. Sound propagation in a liquid containing bubbles has been studied with a set of nonlinear equations postulated by van Wijngaarden in the 1960s [22], and then derived mathematically by Caflisch et al. [21] using Foldy’s method [23]:

(1) $$\frac{\partial p}{\partial t}+\rho_{l}c_{l}^{2}\nabla \cdot u=\rho_{l}c_{l}^{2}\frac{\partial \phi }{\partial t},\;\mathrm{and}$$

(2) $$\rho_{l}\frac{\partial u}{\partial t}+\nabla p=F,$$

where p is the acoustic pressure, u are the velocities, $\rho_{l}$ is the (pure) liquid density, $c_{l}$ is the speed of sound in pure liquid, $\phi =VN=\frac{4}{3}\pi NR^{3}$ is the bubble phase fraction for a bubbly system with a bubble density of N, consisting of identical bubbles each of radius R and V denotes the volume of a single bubble. The bubble density is assumed to be given by a step function:

(3) $$N=\{\begin{array}{c} N_{0}\;if\;\left|P\right|>P_{B}\\ 0\;if\;\left|P\right|\leq P_{B} \end{array},$$

where $P_{B}=1+\sqrt{\frac{4}{27}\frac{S^{3}}{1+S}}$ is the Blake threshold [24,25], with the dimensionless Laplace tension $S=\frac{2\sigma }{p_{0}R_{0}}$, σ is the surface tension between the liquid and gas, $p_{0}$ is the atmospheric pressure, and $R_{0}$ is the equilibrium radius of the bubbles. The momentum source term F can be used to prescribe acoustic velocity sources, e.g., due to the vibrating horn.

The radius of a bubble is obtained by solving a second-order ordinary differential equation (ODE). For an accurate bubble dynamics representation at high forcing pressures, compressibility has to be taken into account. For liquid metals, bubble dynamics can be represented by the Keller–Miksis equation [26]:

(4) $$\rho_{l}\left[\left(1-\frac{\dot{R}}{c_{l}}\right)R\ddot{R}+\frac{3}{2}\left(1-\frac{\dot{R}}{3c_{l}}\right){\dot{R}}^{2}\right]=\left(1+\frac{\dot{R}}{c_{l}}+\frac{R}{c_{l}}\frac{d}{dt}\right)\left[p_{g}+p_{v}-\frac{2\sigma }{R}-\frac{4\mu_{l}\dot{R}}{R}-p_{0}\left\{1-A\sin\left(\omega t\right)\right\}\right],$$

where $p_{v}$ is the vapour pressure of the gas in the bubble, $\mu_{l}$ is the dynamic viscosity of the pure liquid, A is the normalized pressure amplitude (relative to $p_{0}$) and ω is the angular frequency of the ultrasonic source. Taking into account the effect of heat transfer during bubble dynamics [27,28], the gas pressure $p_{g}$ is evaluated by solving the following ODE:

(5) $$\frac{dp_{g}}{dt}=\frac{3}{R}\left[\left(\gamma -1\right)k{\frac{dT}{dr}|}_{r=R}-\gamma p_{g}\dot{R}\right],$$

where k is the thermal conductivity of the bubble gas, T is the temperature inside the bubble and γ is the polytropic exponent. When assuming adiabatic bubble pulsation, the polytropic exponent is given by γ=1.4. The temperature gradient at the bubble surface is approximated linearly following the method of Toegel et al. in water [29]:

(6) $${\frac{dT}{dr}|}_{r=R}=\frac{T-T_{\infty }}{\sqrt{\left(RD\right)/\left\{3\left(\gamma -1\right)\dot{R}\right\}}},$$

where $T_{\infty }$ is the liquid bulk temperature and D is the gas diffusivity. The bubble temperature is solved for by using another ODE expressing the first law of thermodynamics:

(7) $$c_{v}\dot{T}=4\pi R^{2}k\frac{T-T_{\infty }}{l_{th}}-p_{g}\dot{V},$$

where the thermal diffusion length $l_{th}=\min\left(\frac{R}{\pi },\;\sqrt{\frac{RD}{\dot{R}}}\right)$ and $c_{v}$ is the specific heat capacity of the gas.

Solving the Caflisch equations coupled with the above ODEs is very computationally intensive. In general, the acoustic pressure p is required to compute the momentum source term that corresponds to acoustic streaming. To optimize the computational procedure and reduce its time cost, the following approximation is used in recent works.

$ℜ\left(Pe^{i\omega t}\right)$ denotes the harmonic part of the acoustic pressure p. A nonlinear extension [25,30] of the linear Helmholtz equation originally derived by Commander and Prosperetti [31] from the Caflisch equations approximately describes the complex amplitude P as:

(8) $$\nabla^{2}P+K^{2}P=0.$$

Commander and Prosperetti defined the complex wavenumber K using:

(9) $$K^{2}=\frac{\omega^{2}}{c_{l}^{2}}\left(1+\frac{4\pi c_{l}^{2}NR_{0}}{\omega_{0}^{2}-\omega^{2}+2jb\omega }\right).$$

where $\omega_{0}$ is the resonant frequency of the bubbles, j is the complex number satisfying $j^{2}=-1$ and b is the damping factor defined elsewhere [31]. Louisnard [25] generalized this linear model while keeping realistic values for dissipation of energy in inertial cavitation, resulting in a nonlinear model. However, this model suffers from two deficiencies: (1) the real part of $K^{2}$ was taken to approach that of Commander and Prosperetti, and (2) the Helmholtz equation used by the model comes from the linear theory. To address these issues, the real and imaginary parts of the coefficient $K^{2}$ have been rigorously re-derived by Trujillo [30] as:

(10) $$ℜ\left(K^{2}\right)=\frac{\omega^{2}}{c_{l}^{2}}-\frac{A}{\left|P\right|},\;\mathrm{and}$$

(11) $$ℑ\left(K^{2}\right)=-\frac{B}{\left|P\right|},$$

where

(12) $$A=-\frac{\rho_{l}\omega^{2}}{\pi }\int_{0}^{2\pi }\frac{\partial^{2}\phi }{\partial \tau^{2}}\cos\left(\tau +\frac{\pi }{2}\right)d\tau ,$$

(13) $$B=\frac{\rho_{l}\omega^{2}}{\pi }\int_{0}^{2\pi }\frac{\partial^{2}\phi }{\partial \tau^{2}}\sin\left(\tau +\frac{\pi }{2}\right)d\tau ,\;\mathrm{or}$$

(14) $$A=-\frac{\rho_{l}\omega^{2}}{\pi }\int_{0}^{2\pi }\frac{\partial \phi }{\partial \tau }\sin\left(\tau +\frac{\pi }{2}\right)d\tau ,$$

(15) $$B=-\frac{\rho_{l}\omega^{2}}{\pi }\int_{0}^{2\pi }\frac{\partial \phi }{\partial \tau }\cos\left(\tau +\frac{\pi }{2}\right)d\tau ,$$

where the non-dimensional time τ is within one period, i.e., [0, 2π]. The boundary conditions for the nonlinear Helmholtz equation are generally defined as:

- ∇P·n=0 for infinitely hard boundaries (such as crucible walls);
- $P=AP_{0}$ at the surface of the sonotrode;
- Setting P=0 in the cell layer above the liquid level to approximate the π phase shift that occurs upon reflection from the free surface [32].

This nonlinear Helmholtz equation is rather simple to solve using the finite element method (FEM), thereby facilitating the numerical evaluation of the acoustic field in the presence of cavitation bubbles [19,20,25,30] in commercial packages such as COMSOL Multiphysics. The solution of this equation using the finite volume method (FVM) is trickier and requires special preconditioning [32]; however, the flow equations are simpler to solve in the FVM framework.

### 2.2. Macroscopic Flow Model

Acoustic streaming models in the literature generally follow the work of Eckart [33] by incorporating the streaming force f as:

(16) $$f=-\nabla \left(\rho_{l}\bar{v⊗v}\right),$$

where v is the acoustic velocity to the continuity and momentum conservation equations, leading to:

(17) $$0=\nabla \cdot \left(\rho_{l}u\right)+\nabla \cdot \left(\bar{\rho_{v}v}\right),$$

(18) $$0=f-\nabla p+\mu_{l}\nabla^{2}u,$$

where $\rho_{v}$ is the density variation that is caused by the primary acoustic field. However, Equation (18) is the momentum equation of a creeping flow driven by the acoustic streaming and is therefore applicable to Reynolds numbers much smaller than 1 [20]. Since acoustic cavitation processes would involve much larger Reynolds numbers, the streaming velocity should instead be calculated from a full steady-state Navier–Stokes equation [34]:

(19) $$\nabla \left(\rho_{l}u⊗u\right)=f-\nabla p+\mu_{l}\nabla^{2}u.$$

However, the solution of Equation (19) is difficult since the streaming flow observed experimentally is turbulent [35], and resolving small-scale eddies with the Navier–Stokes equation is not trivial [20]. Instead of solving for streaming directly, the latest papers follow the approach of Louisnard [20] by computing the streaming force from the solution of the nonlinear Helmholtz Equation (8) and injecting the result into the momentum equation. This approach has been validated in recent works describing acoustic streaming [20,35,36,37] and has also been applied to DC casting [38].

## 3. Numerical Simulations of the Acoustic Field in Crucibles, Moulds and Launders

There is a dearth of contributions in the literature regarding the specific modelling of ultrasonic melt processing. This is not surprising since accurate measurements of acoustic pressures in aluminium have only recently been made available [39,40]. Some of the first modelling contributions are those of Nastac [41,42,43], who presented two approaches for modelling grain refinement of an A356 alloy. A similar approach is followed by other authors to model nanoparticle dispersion [44] and the distribution of acoustic pressure in a launder [45]. The first method consists of solving the Reynolds-Averaged Navier-Stokes (RANS) equations using a classical hydrodynamic cavitation model [46] that is implemented in commercial Computational Fluid Dynamics (CFD) packages. The essence of this method can be summarized as follows. The liquid–bubble mass transfer is governed by a bubble transport equation in the following form:

(20) $$\frac{D\left(\rho_{b}\phi \right)}{Dt}=R_{G}-R_{C},$$

where $\rho_{b}$ is the bubble density and the source terms $R_{G}$ and $R_{C}$ account for bubble growth and collapse, respectively. These source terms are calculated using the growth of a single spherical bubble based on a bubble dynamics model (e.g., Rayleigh–Plesset [47], Keller–Miksis [26], Neppiras–Noltingk [48]). This equation is coupled with the flow conservation equations, together with a suitable closure for turbulence. However, as beautifully as it is presented by Louisnard [20], this model is restricted to bubbly liquids containing vapour bubbles only. With the vapour pressure of aluminium at the melting point being negligible [49], it is unlikely that aluminium vapour bubbles would form in the melt bulk [50] with gas, hydrogen-filled bubbles, forming instead. This therefore, prohibits the use of any hydrodynamic cavitation models for the inertial acoustic cavitation bubbles that are present in liquid aluminium treatment.

The second approach in Nastac’s contribution is, however, more appropriate and is a precursor to the method highlighted in this overview. In this indirect method, the acoustic field is solved by using a linear Helmholtz equation, closed with the Neppiras–Noltingk model [48]. However, as argued in the previous section, this method suffers from various deficiencies: the linear Helmholtz model is inadequate in the presence of cavitation bubbles, since pressure propagation is nonlinear in this regime. The Neppiras–Noltingk model does not account for acoustic radiation, which is crucial at high forcing pressures. Other authors also used a linear acoustic propagation model to study the treatment of AlSi7Mg alloy melt in sand casting, even though pressures larger than 2 MPa have been predicted [51]. A linear model was also employed to compute the acoustic pressure field in a SCN-1 wt% camphor alloy, which is often used as a transparent analogue to aluminium melt [13,52].

**Figure 1:**
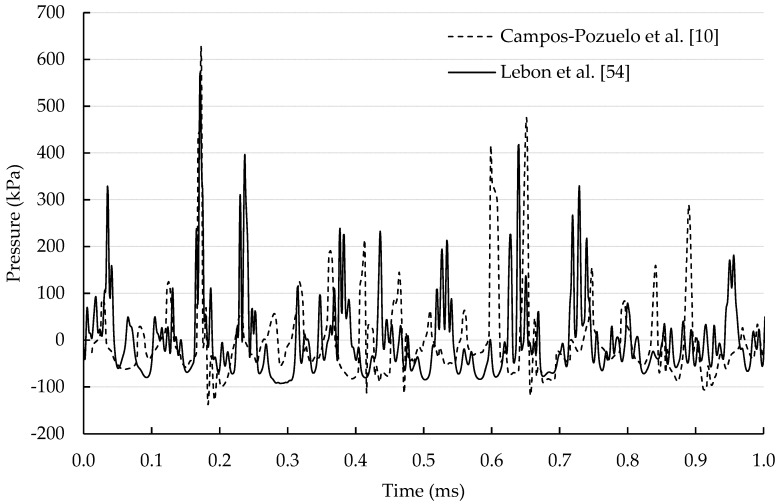
Validation of Caflisch approach to computing acoustic pressures [54] using experimental data from Campos-Pozuelo et al. [10].

Another attempt to obtain an accurate prediction of the acoustic pressure field was through the solution of the Caflisch equations (Equations (1) and (2)) [38]. In this approach, Lebon et al. directly computed the acoustic field using the nonlinear equations governing sound propagation in bubble liquids [21,53,54] and validated the model using experimental data from the literature as shown in Figure 1.

Adequate pressures were predicted as compared with the measurements using a calibrated cavitometer [39,40] enabling the extension of the model to account for nanoparticle deagglomeration [55], fragmentation of dendrites [56], the erosion of thermally-sprayed splats [57] or contactless ultrasound due to Lorentz forces from an electromagnetic coil [58]. However, this method suffers from various drawbacks. A bubble dynamics ODE must be solved in each computational cell of the domain, with the use of an adaptive time-stepping scheme to stabilize the solution procedure for each computational cell. The method is also prone to numerical diffusion and requires the use of high-order discretisation schemes in space and time, and a special staggering scheme [59]. These issues prohibit the application of the model to complex 3D geometries due to the extreme computational requirements.

**Figure 2:**
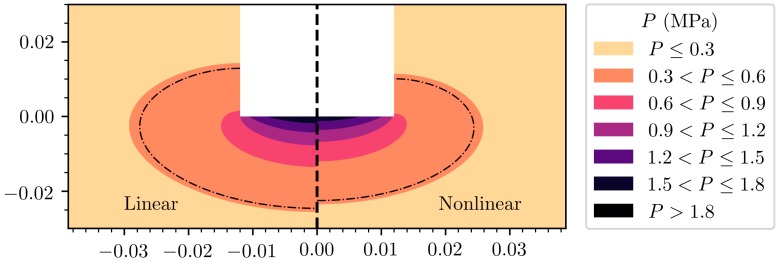
The difference in predicted acoustic pressures between linear and nonlinear models. The nonlinear model includes the attenuating effect of cavitating bubbles below the sonotrode. The dash-dotted line denotes the Blake threshold for hydrogen bubbles in aluminium, with $R_{0}=3$ µm.

More recently, nonlinear models of pressure propagation have been used in the context of melt processing. Nonlinear models are required to more adequately capture the attenuation of pressure due to the presence of cavitating bubbles as shown in Figure 2. Huang et al. [60] used an improved nonlinear Helmholtz model [61] to predict the cavitation depth in sonication of an AlCu melt. Lebon et al. used Louisnard’s model to compute acoustic pressures in water and aluminium vessels [35]. This model was adapted and improved to model acoustic pressures in DC casting [38]. The same approach has also been used by Yamamoto and Komarov [37]. The last two studies are conducted in conjunction with acoustic streaming and are discussed in the next section.

## 4. Effect of Acoustic Streaming

The numerical study of acoustic streaming in melt processing is even sparser in the literature. A prediction of ultrasonic DC clad casting using the Navier–Stokes equations only has been recently published [62]. Acoustic streaming is implemented by a direct solution of the sonotrode motion in ANSYS Fluent and applying the transient effects in steady-state equations, although the implementation details of the process are not described. The model does not include the effect of cavitation bubbles, so acoustic shielding is not considered. As reported in [63], this method does not appear to capture the flow reversal observed at certain irradiation powers.

Other significant contributions, however, employ acoustic streaming models computing the acoustic streaming force as per Equation (16). Simulation of convective flow for an Al-2% Cu alloy has been performed by Wang et al. [12,64,65] using the Lighthill approach [34]. This approach predicts a fast velocity jet below the sonotrode, and comparison with a corresponding experiment reveals that the fast streaming flattens the temperature gradient and promotes an equiaxed grain structure. Another approach involved the use of the Ffowcs Williams and Hawkings (FW—H) equation generally used to compute the propagation of aerodynamic noise to model the acoustic pressure in Fluent [66,67]. Another commercial CFD software package, Flow3D, has been used to model acoustic streaming during the treatment of an A356 alloy melt without detailing the modelling procedure [68].

**Figure 3:**
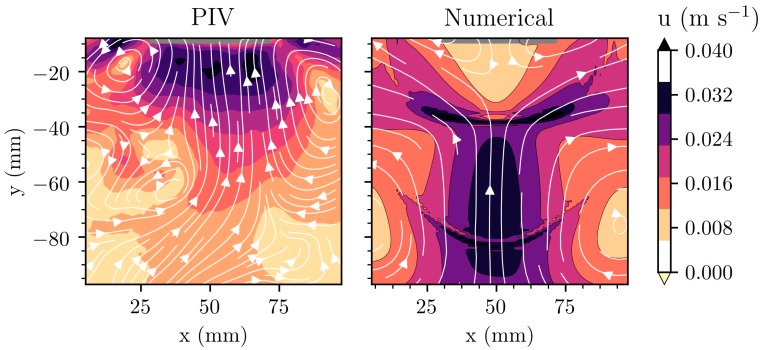
Comparison between measured velocities using particle image velocimetry (PIV) [63] in water and predictions of acoustic streaming using the numerical model described in [35]. The velocities are in m s^−1^. The grey bar at the top of each contour represents the vibrating surface. The dataset used to reproduce these results is available elsewhere [69].

Inspired by Louisnard’s nonlinear model coupled with acoustic streaming, Lebon et al. [35] validated an acoustic streaming model using results from a particle image velocimetry (PIV) experiment using a TSI system [63] as shown in Figure 3. While the model offers only qualitative agreement with the experiment, this progress is encouraging because:

1. Despite simulating the problem in two dimensions, the correct order of magnitude of acoustic streaming is recovered.
2. The net flow reversal below the sonotrode observed at low operating powers is predicted by the model.
3. The comparison holds for a time-averaged analysis of transient results, which mimics the way velocities are recorded by PIV.
4. The model is tractable since the Helmholtz equation is easier to solve than a system of ODEs representing bubble dynamics.

However, a remaining challenge is the suitable choice of the bubble number density as a parameter for the simulation. This study of acoustic streaming in water revealed that the predicted flow field is sensitive to the assumed bubble volume fraction, and therefore of bubble density by extension. The bubble density can also vary spatially, although a step function such as the one described by Equation (3) helps in limiting the presence of bubbles in regions below the Blake threshold. Semi-empirical values in the range 10^9^–10^12^ m^−3^ have been reported to lead to acceptable results [32,35,54,70,71,72]. Aside of the difficulties in choosing the appropriate bubble density for a particular simulation, variation in bubble density due to differences in melt quality, presence of impurities and agglomerates or variation in degassing times presents further challenges.

**Figure 4:**
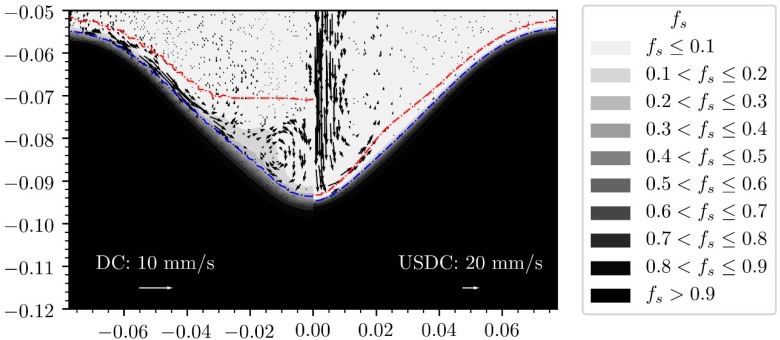
Comparison of sump profiles between conventional DC casting (left) and ultrasonic-assisted DC casting (right). $f_{s}$ is the solid fraction using the casting conditions defined in [38]. Arrows are shown for the scale of the velocity field. The red dash-dotted line represents the liquidus temperature and the blue dash-dotted line denotes the coherency temperature (solid packing fraction). The dataset used to reproduce these results is available elsewhere [73].

**Figure 5:**
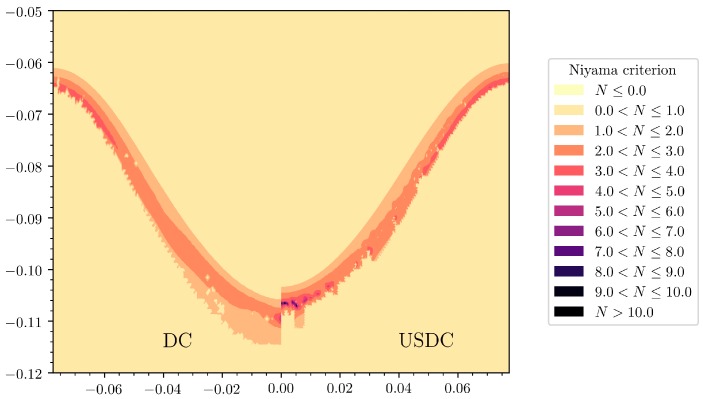
Comparison using the Niyama criterion between conventional DC casting (left) and ultrasonically assisted DC casting (right) using the casting conditions defined in [38]. The larger values upon sonication indicate an increased probability of porosity defects at the centre of the cast billet.

This model was further improved using Trujillo’s mathematically rigorous derivation of the complex wavenumber (9) and was then applied to DC casting using a continuum model [38]. Figure 4 shows the results of a numerical model of ultrasonic processing in DC casting. The predicted sump profile is altered by the fast streaming jet of hot liquid aluminium down the axis of the billet, thereby shortening the transition region at the centre of the billet. Observed grain orientations at the centre of cast billets confirm this prediction [38]. The model also predicts a slightly increased rate of porosity defects at the centre of billet cast with ultrasonic processing, as shown by the increased Niyama criterion at the centre of the domain (Figure 5). However, any experimental observation of increased porosity has not been reported so far.

Another research group independently used the same acoustic pressure formulation: Yamamoto and Komarov applied a similar model to aluminium [37] to reveal that attenuation of ultrasound and wavenumbers are larger in the molten aluminium than in water and that acoustic streaming flow is slower in the aluminium melt as compared with water. This is in agreement with our experimental study in [40], where shielding and acoustic damping were found to be more pronounced in liquid aluminium compared to water, obstructing the wave propagation into the bulk. 

## 5. Current Challenges and Future Outlook

This summary has highlighted the requirement of considering the appropriate physics when modelling the complex phenomenon of ultrasonic melt treatment. However, more effort is required for an accurate prediction of actual treatment conditions. Aside from a recent contribution studying resonance in crucibles [58], the boundary conditions used in the models encountered in USP modelling so far are basic and do not take into account the vibration of the solid walls of a sonoreactor or reflection off real, rough crucible walls. Crevices in the walls could also act as seeds for nucleating bubbles and these are not taken into account by any model encountered so far. This is crucial in situations where resonance is required and both the changing bubbly media and imperfect container walls affect the resonant frequency of the system [58,74].

The stability of the heat balance solver upon mesh deformation and solidification front motion is still an issue for accurate modelling of ultrasonic processing in the presence of solidification. This limits the accuracy of casting simulations and need to be addressed for more accurate sump profile predictions. There is also uncertainty on the effect of the entrained cavitating bubbles and acoustic streaming jet on the packing fraction in the semi-solid region of a casting domain, and therefore on the delimitation between slurry and mushy zones. These need to be quantified accurately for more reliable predictions.

Since acoustic streaming modifies the grain morphology of the billet, the coherency temperature is expected to vary locally in the sump. Further study is required to determine the dependency of this parameter on the flow. However, an accurate a priori prediction is rendered difficult since the knowledge of the grain size and morphology is required before choosing the correct solid packing fraction.
